# Year of Birth Effects in the Historical Decline of Tuberculosis Mortality: A Reconsideration

**DOI:** 10.1371/journal.pone.0081797

**Published:** 2013-12-11

**Authors:** Romola J. Davenport

**Affiliations:** Department of Geography, University of Cambridge, Cambridge, United Kingdom; Fondazione Bruno Kessler, Italy

## Abstract

Birth cohort patterns in mortality are often used to infer long-lasting impacts of early life conditions. One of the most widely accepted examples of a birth cohort effect is that of tuberculosis mortality before the late 1940s. However the evidential basis for claims of cohort-specific declines in tuberculosis mortality is very slight. Reanalysis of original or enhanced versions of datasets used previously to support claims of cohort effects in tuberculosis mortality indicated that: 1. where the initial decline in tuberculosis mortality occurred within the period of observation, onset of decline occurred simultaneously in many age groups, in a pattern indicative of ‘period’ not cohort-dependent effects. 2. there was little evidence of ‘proportional hazard’-type cohort patterns in tuberculosis mortality for any female population studied. Therefore any mechanisms proposed to underlie this type of cohort pattern in male mortality must be sex-specific. 3. sex ratios of tuberculosis mortality at older ages peaked in cohorts born around 1900, and resembled cohort sex ratios of lung cancer mortality. This analysis indicates that age-specific patterns in the decline in tuberculosis mortality before 1950 are unlikely to reflect improvements in early life conditions. The patterns observed are generally more consistent with the influence of factors that reduced mortality simultaneously in most age groups. Additional influences, possibly smoking habits, impeded the decline of tuberculosis in older adult males, and produced the sex-specific shifts in age distributions of mortality that were previously interpreted as evidence of cohort-dependent mortality decline.

## Introduction

Birth cohort effects in epidemiology can be defined as variations in risk according to year of birth and arising from influences unique to particular birth cohorts, or which affected some birth cohorts more than others (for example because the effect varied with age at exposure) [1]. A key area of interest with respect to cohort effects is the influence of childhood experience on adult survival. However the impact of early life events on mortality in later life remains highly contested (e.g. [2]–[6]). In an influential review of early life effects, Elo and Preston concluded that a number of childhood health conditions carried implications for survival at older ages, “but only in the case of respiratory tuberculosis has the demographic importance of a specific mechanism been established by cohort studies” [7]. Numerous authors have reported birth cohort patterns in tuberculosis mortality decline (Table 1), and these patterns have generally been interpreted as evidence of progressive improvements in early life conditions, that increased the survival of successive cohorts at all ages. Cohort patterns in mortality from respiratory tuberculosis in the late nineteenth and early twentieth centuries are often used to exemplify cohort effects in epidemiology textbooks, and have been proposed to underpin the cohort patterns observed in historical all-cause mortality decline in England and Wales [2], [7]. Indeed, the term ‘cohort’ was first used in the demographic sense of a birth cohort in the context of tuberculosis mortality [8].

**Table 1:** Tuberculosis mortality data used in this and previous studies of cohort effects in tuberculosis.

population	type and period analysed in thispaper (with source)	right shift in age ofpeak period mortality	decline underway	references reportingcohort patterns
Australia	all TB, 1907–1980 [40]	males	yes	[76]
Danish towns		no	yes	[22], [77]
Denmark		not shown	yes	[20], [21]
England & Wales	respiratory TB, 1848–1980 [36], [37]	males	yes	[8], [20]–[23], [27], [34], [49], [53], [77]–[80]
France	all TB, 1925–1980 [46]	males	yes?	
Ireland	all TB, 1871–1941 [22]	no	no	[22]
Italy		no	yes	[34]
Massachusetts	respiratory TB, 1880–1950 (this study)	males	yes	[8], [22], [25], [77]
Japan	respiratory TB, 1950–1980 [47]			
New Zealand		males	yes	[34]
Norway	respiratory TB ages 0–14, all TB ages15+, 1871–1955 [72]	No	no	[20]–[22], [80]
Ontario		males	yes	[81]
Paris		males	yes	[22], [52]
Scotland	all TB, 1861–1941 [22]	males	no	[22]
Sweden		males	yes	[20]–[22], [77]
Switzerland		males	yes	[82]
US death registration states	all TB, 1900–1980 [49]	yes	yes	[8], [25], [83]

**Figure 1:**
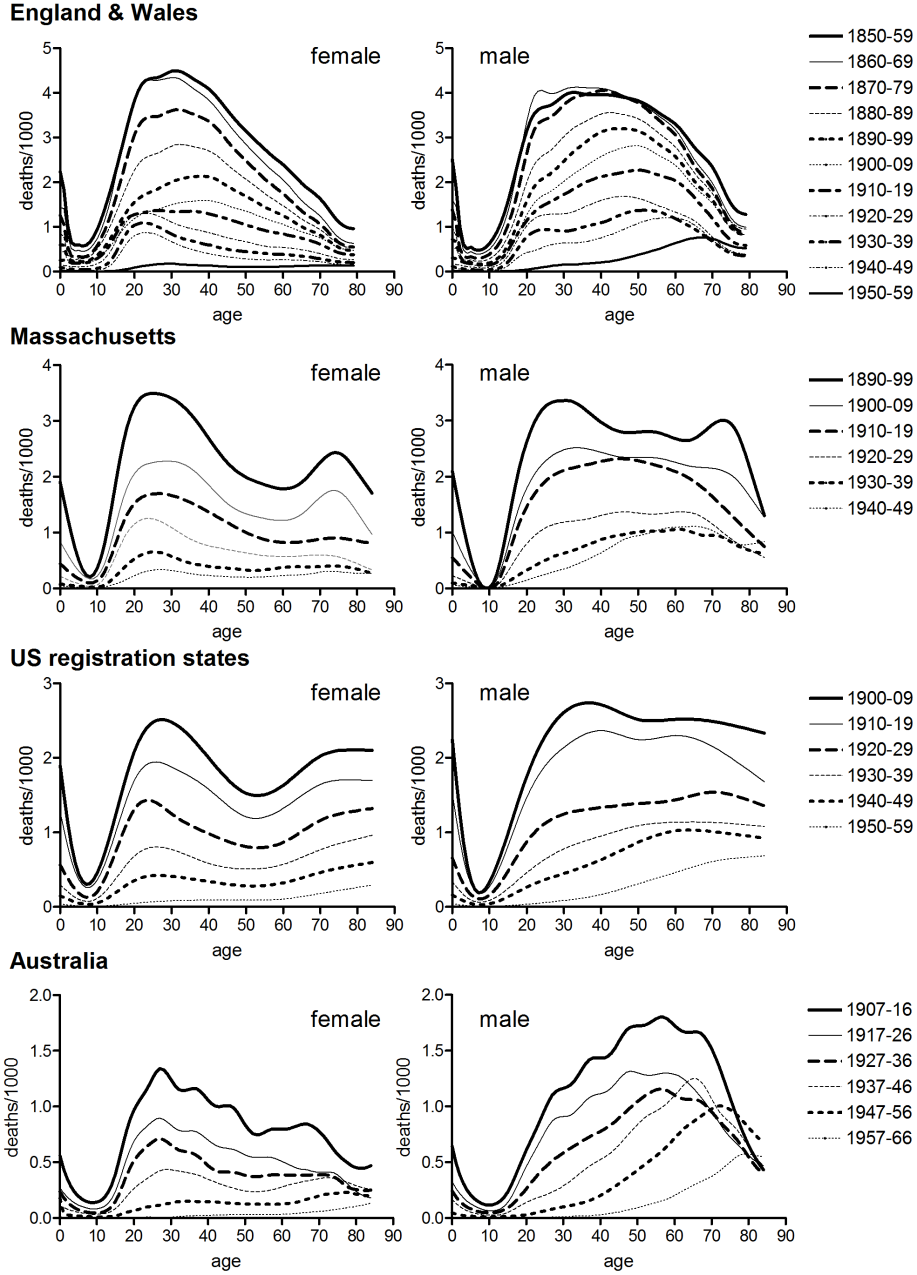
Age-specific tuberculosis mortality by period of death. Data refer to annual rates of respiratory tuberculosis (England & Wales, and Massachusetts), or all tuberculosis (US death registration states, Australia).

Tuberculosis is a chronic infectious disease with a complex and poorly understood aetiology that provides rich possibilities for life course influences. Disease can arise from primary infection or re-infection, or from re-activation of earlier infections that have persisted in a latent form. The relative contributions of recent and prior infection events to adult disease remain contested, and may vary with transmission rates [9]–[12]. In high prevalence settings first infection commonly occurs in childhood and may result in disease progression or in a successful immune response leading to elimination or sequestration of the bacilli in latent form. The protective effects of childhood infection and immunity (or BCG vaccination; [13]) appear to wane in late adolescence and disease in adolescence and adulthood may reflect re-infection or the reactivation of childhood infections. Disease in childhood is associated mainly with disseminated forms of disease such as miliary tuberculosis and tubercular meningitis, whereas tuberculosis in adulthood is predominantly a respiratory phenomenon [14]. Historically, respiratory tuberculosis mortality was characterised by a distinctive bimodal age pattern, with peaks of clinical illness and mortality in infancy and early adulthood (Figure 1). In addition, historical age patterns of tuberculosis mortality showed clear differences by sex. Females experienced higher mortality rates than males in adolescence and early adulthood (ages 10–39), and when tuberculosis mortality was concentrated at these ages then the overall sex ratio of mortality from tuberculosis reflected a heavier toll amongst females. However, as tuberculosis mortality declined in the period before antibiotic treatment males generally experienced slower rates of improvement at ages 40 and older, and by the mid-twentieth century tuberculosis mortality in most now-developed populations was characterised by a large male excess. Tuberculosis mortality dropped precipitously with the introduction of effective chemotherapy in the mid-1940s and the widespread adoption of BCG vaccination in the 1940s and 1950s. The peculiar historical age distribution of tuberculosis, with peaks of mortality in infancy and early adulthood, disappeared, and mortality became concentrated in late adulthood in both sexes in now-developed country populations. Recent sex and age patterns of tuberculosis mortality in high prevalence populations reflect in part the association of tuberculosis with HIV infection, and possibly sex-specific under-reporting, but still show an older age distribution than was typical of tuberculosis mortality before the antibiotic era [15], [16].

### Cohort Patterns in Tuberculosis Mortality

The Norwegian physician Andvord is usually credited as the first to have recognized cohort patterns in tuberculosis data in the 1920s [17], although Brownlee described ‘generational’ patterns in tuberculosis mortality in England and Wales as early as 1916 [18], [19]. Andvord argued that improvements in tuberculosis mortality appeared first in the youngest age groups, and then with progressively longer lags in older age groups [20], [21]. He used (all-sex) Norwegian, Swedish, Danish and English data to argue that there had been a progressive shift in the peak age of tuberculosis mortality in adults from early adulthood to older ages over time. However when mortality was plotted by year of birth then the age pattern of tuberculosis mortality was the same for all birth cohorts, with a constant peak in early adulthood. That is, the difference between successive cohorts was only in the level of tuberculosis mortality, with each successive generation experiencing a fall in risk that was proportional at each age. Thus although tuberculosis mortality appeared to rise with age in cross-sectional (‘period’) data in the twentieth century, in fact older adult victims were survivors of birth cohorts that had experienced even higher rates of tuberculosis mortality in young adulthood. Andvord hypothesized that susceptibility to respiratory tuberculosis was acquired or determined early in life, and infection rates in childhood determined the level of mortality for each birth cohort at older ages, because the age pattern itself was “almost ‘predestined’” [20].

The type of cohort pattern described by Andvord can be termed a ‘proportional hazard’ cohort pattern, because the proportional difference in mortality rate for a given cohort compared to other cohorts was the same at all ages. Andvord’s findings were popularized by Frost, who discovered similar proportional hazard type cohort patterns in tuberculosis mortality in the United States and Massachusetts [8]. This type of cohort pattern was subsequently detected by a number of other workers in various populations (Table 1), and was analysed most extensively by Springett [22]–[24]. Springett was only able to demonstrate proportional hazard-type cohort patterns in males in England and Wales, Scotland and Danish towns, and did not find evidence of such patterns in the male populations of Ireland, Norway, Sweden, and Denmark, or for females in any of the populations he examined. Nevertheless he argued that the decline of tuberculosis mortality was best interpreted as a cohort phenomenon, originating in a reduction in infection of young adults that reduced the risk of reactivation of latent infection at later ages in surviving members of the cohort [22]–[24]. Mason and Smith analysed male death rates from tuberculosis in Massachusetts and the USA, and observed that “until the advent of effective chemotherapy, successive cohorts moved through life as though they had different probabilities of dying by tuberculosis assigned at birth” [25]. Cohort patterns have also been reported in age-specific notification rates or prevalence in the second half of the twentieth century [26]–[29].

The evidence for proportional hazard-type cohort patterns in historical tuberculosis mortality rests principally on two types of evidence: (1) the regularity of age patterns of tuberculosis mortality by birth cohort, compared with age patterns in period (cross-sectional) data; and (2) the progressive shift of the age distribution of tuberculosis mortality to older ages observed in some male populations. In both cases the evidence is circumstantial, and is limited to males, since age patterns were fairly stable by both cohort and period in female populations analysed, implying similar rates of decline at all adult ages [19], [22], [24]. Although sex differences in the age patterns of tuberculosis mortality were a major area of interest throughout the first half of the nineteenth century (e.g. [30]–[32]), the lack of evidence for cohort patterns in female tuberculosis rates was rarely investigated (but see [24] for an attempt), and females were sometimes excluded from analysis (e.g. [18], [25]).

In addition to graphical analyses, age-period-cohort models were used to detect period and cohort patterns in three studies [25], [33], [34]. Sacher and Mason and Smith applied age-period-cohort models to tuberculosis mortality data from the same population (Massachusetts) with very different results, emphasizing what Mason and Smith termed the ‘fragility’ of the modelling process, that is the sensitivity of their results to the choice of assumptions required to fit the models. Sacher assumed that cohort effects were negligible for early birth cohorts, and found that period effects declined monotonically across the period of observation [33], whereas Mason and Smith assumed age effects were similar for age groups 0–9 and 10–19, and found negligible period effects and strong cohort effects (although their model was less successful in describing patterns for US males) [25]. Another issue in the application of age-period-cohort models to historical tuberculosis mortality is the question of whether the age pattern of tuberculosis mortality was fixed, at least before effective chemotherapy. If a fixed age pattern is assumed then the observed changes in the age pattern of mortality in some male populations over time are most readily interpreted as cohort patterns. If however the age pattern is allowed to vary, then this requires the inclusion in the model of new variables or interactions between age and period or cohort for which there may be little evidential basis. While a biologically determined age pattern of mortality may be applicable for some diseases [35], the variations in age patterns of tuberculosis mortality by sex and between populations caution against this assumption, as Mason and Smith note [25]. Given the apparent complexity of factors influencing historical patterns of tuberculosis mortality, age-period-cohort models are potentially useful for data exploration, but are unsuited to hypothesis-testing. Instead the present approach relied on testing for the presence of certain features deemed by previous authors to be diagnostic of cohort patterns in tuberculosis decline, such as evidence of age-lagged onset or acceleration of tuberculosis mortality declines.

Close analysis of cohort patterns was precluded in previous studies by the use of occasional years or aggregated series of years of data, which prevented the construction of well-defined birth cohorts and the analysis of continuous series of rates. The present study is the first to use single calendar years of cause-specific age-specific mortality rates for analysis of cohort patterns in tuberculosis mortality in nineteenth century and early twentieth century populations. A sustained comparison of sex differences in tuberculosis mortality is also presented. A graphical approach was employed to provide simple tests for the presence of unambiguous proportional hazard-type cohort patterns. The very considerable problem of misdiagnosis of tuberculosis is ignored, because the purpose of the study was to test the existence of cohort patterns in *recorded* historical tuberculosis mortality using the same sources deployed by earlier authors.

## Methods

Annual series of sex-specific age-specific tuberculosis mortality data were obtained as counts of deaths for England and Wales (Economic and Social Data Service SN5705; [36], [37]), and Massachusetts [38] (available as Data S1, S2), and as death rates for the United States death registration area [39] and Australia [40]. Sources of tuberculosis mortality rates for other populations are given in Table 1. Annual population estimates by age were obtained for England and Wales from the Human Mortality Database [41] and constructed by geometric interpolation from decennial federal census data for Massachusetts [42]. Annual age-specific mortality rates for five or ten year age groups were interpolated to single year of age rates using cubic spline fitting. For England and Wales rates were converted to deaths, and deaths and populations divided between years to construct age-specific mortality rates by single years of birth and age. These were then converted to cause-specific q-type rates using formula 2 of Preston et al. [43] to give probabilities of dying of tuberculosis within specified age intervals, calculated using single year of age mortality rates and populations at risk. This method assumes that the mortality rate (m) approximates the force of mortality (µ) for a given cause over small age intervals, a reasonable assumption for adults and for single year of age mortality rates. For other populations no reliable estimates of populations by single years of age existed for the periods required. In these cases cohort q-type rates were constructed from period rates by the same method on the assumption that the cohort year of birth = year of death – age at death.

Changes in the age distribution of tuberculosis mortality were measured as changes in the median age at death. To calculate median age at death age-specific mortality rates were applied to a common population standard, the 1901 population of England and Wales (both sexes) [41], to generate counts of deaths by age for each population that were comparable between populations and unaffected by the age structure of each population (a technique known as ‘indirect standardisation’). Median age at death was calculated using standardised deaths for ages 10 to 79, to avoid potential effects of changes in rates of misdiagnosis in childhood and errors caused by age misreporting or aggregation at oldest ages. Median age at death by birth cohort could only be calculated for a series of years in the case of England and Wales, because the calculations required a minimum of 70 years of data for each birth cohort and the data series for other populations were too short. Trends in median age at death were assessed by the Mann-Kendall trend test (a non-parametric test for time trends) [44].

Contour diagrams were constructed using Lexis 1.1 software [45], and the annual rate of improvement in age-specific death rates was plotted using the derivative of logged annual period death rates after smoothing with cubic splines. Respiratory tuberculosis rates were used where possible, because the decline of respiratory tuberculosis followed a different time course from non-respiratory tuberculosis, and the two categories had different age distributions. Non-respiratory tuberculosis constituted a very small proportion of all tuberculosis deaths in adults. Lung cancer rates were obtained from the same sources as tuberculosis data for England and Wales, Australia and France [46], and from the WHO mortality database for Japan and the USA [47]. All-cause mortality rates were obtained from the Human Mortality Database [41]. Cohort rates were constructed as for tuberculosis. Data were analysed using STATA 9.1 software and XLSTAT (for Mann-Kendall trend analyses).

## Results

**Figure 2:**
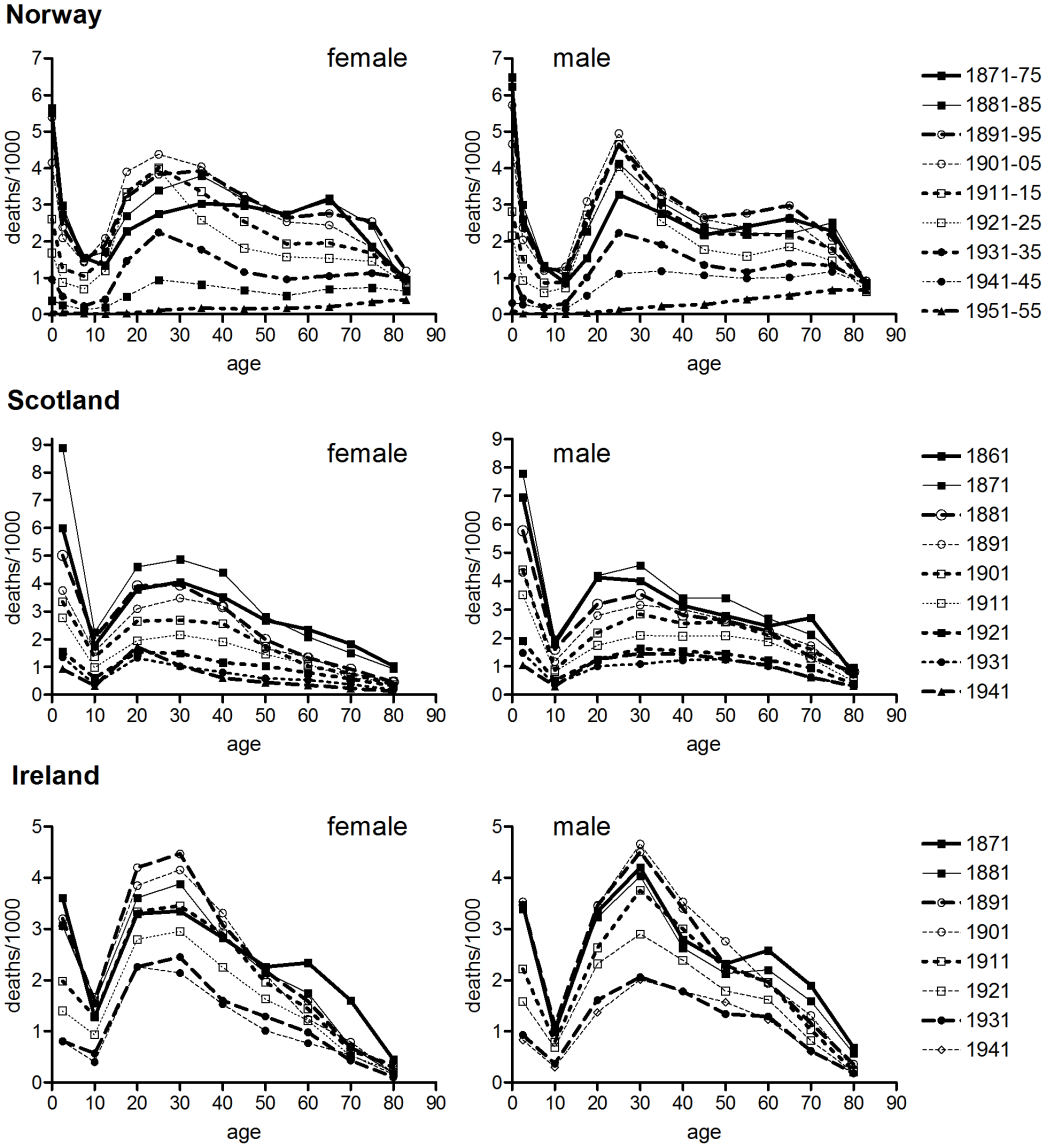
Age-specific tuberculosis mortality by period of death. Data refer to five yearly rates of respiratory tuberculosis ages 0–14, all tuberculosis ages 15+ (Norway), or single years of data at decadal intervals, all tuberculosis (Scotland, Ireland).

**Figure 3:**
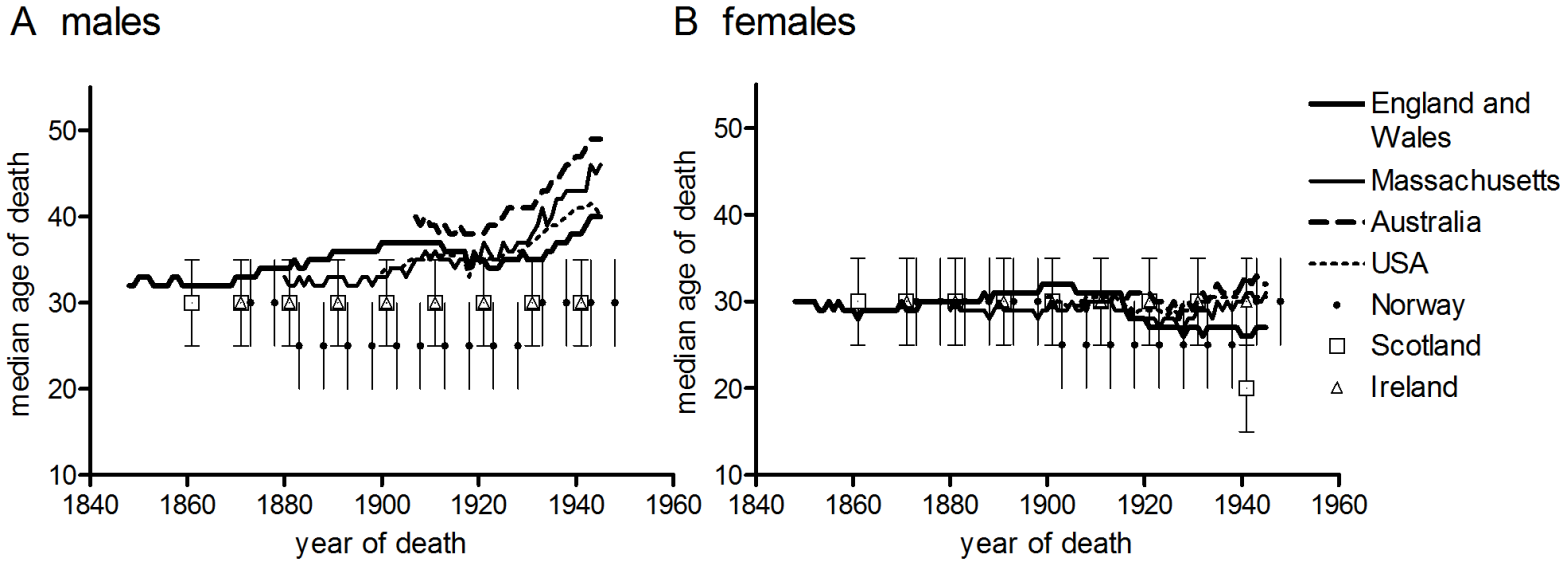
Median age at death from tuberculosis, by year of death. Calculated by indirect standardisation of deaths at ages 10–79 years.

Figures 1, 2 and 3 confirm the differences in historical tuberculosis mortality by population, age and sex observed in previous work. Figures 1 and 2 show the age distribution of tuberculosis mortality in seven populations for which relatively long-run annual cause of death records were available. In the case of England and Wales, Massachusetts, the USA and Australia there was a progressive shift in the age distribution to older ages in males over the first half of the twentieth century. However in the male populations of Norway, Scotland and Ireland there was little evidence of a shift to the right, although there was a reduction in the relative size of the peak in early adulthood. Norway was notable in that the age distribution was younger for males than for females, and male rates showed little shift to the right before the antibiotic era, despite Andvord’s claims [48]. By contrast with males, shifts in the female age distributions were temporary and mortality remained concentrated in early adulthood (US females are an exception, but even in this case the peaks of the trimodal distribution did not shift, contrary to the progressive shifts predicted with cohort effects).

Shifts in the age distribution of tuberculosis mortality could be quantified using median age at death. Figure 3a demonstrates the progressive right shift (indicated by a rising trend in median age) in the age distribution of tuberculosis mortality for the male populations of England and Wales, Massachusetts, the USA and Australia. It also confirms the relative stability (and youthfulness) of age distributions for male populations of Norway, Scotland and Ireland. In stark contrast with the heterogeneity of age distributions and trends between male populations, Figure 3b demonstrates the relative uniformity of age distributions over time and between populations for females of all populations.

The existence of time trends in median age at death was tested statistically using non-parametric Mann-Kendall tests (Irish, Norwegian and Scottish data were excluded from these analyses because only data for broad age groups were available). The median age at death rose significantly in the male populations of England and Wales, Massachusetts, the USA and Australia, although the rate of rise varied from 0.06 years of age per calendar year (for the male population of England and Wales) to 0.3 years per year (for Australian males). Although Mann-Kendall tests identified trends in the two of the four female populations tested (England and Wales and Australia) the estimated rate of change in median age was slight compared with male trends, and negative in the case of females in England and Wales (Table 2). Consistent with Spicer’s findings [49] evidence of a sustained right shift in the age distribution of tuberculosis (considered indicative of cohort patterns) was found in only some male populations, and was absent or negligible in all female populations tested.

**Table 2:** Mann-Kendall tests^1^ for trend in median age at death from tuberculosis (ages 10–79).

Population	Sex	Period	Minimummedian age	Maximummedian age	Kendall’s tau	P (slope = 0)	Sen slope (year of age per calendar year)
England and Wales	male	1848–1945	32	40	0.64	<0.001	0.06
Massachusetts	male	1880–1945	32	46	0.85	<0.001	0.15
USA	male	1900–1945	33	41	0.81	<0.001	0.17
Australia	male	1907–1945	38	49	0.75	<0.001	0.30
England and Wales	female	1848–1945	26	32	−.021	0.005	−0.02
Massachusetts	female	1880–1945	27	31	0.04	0.639	0
USA	female	1900–1945	28.5	30.5	0.11	0.325	0
Australia	female	1907–1945	28	33	0.35	0.005	0.03

^1^ Least-squares linear regression produced very similar results.

**Figure 4:**
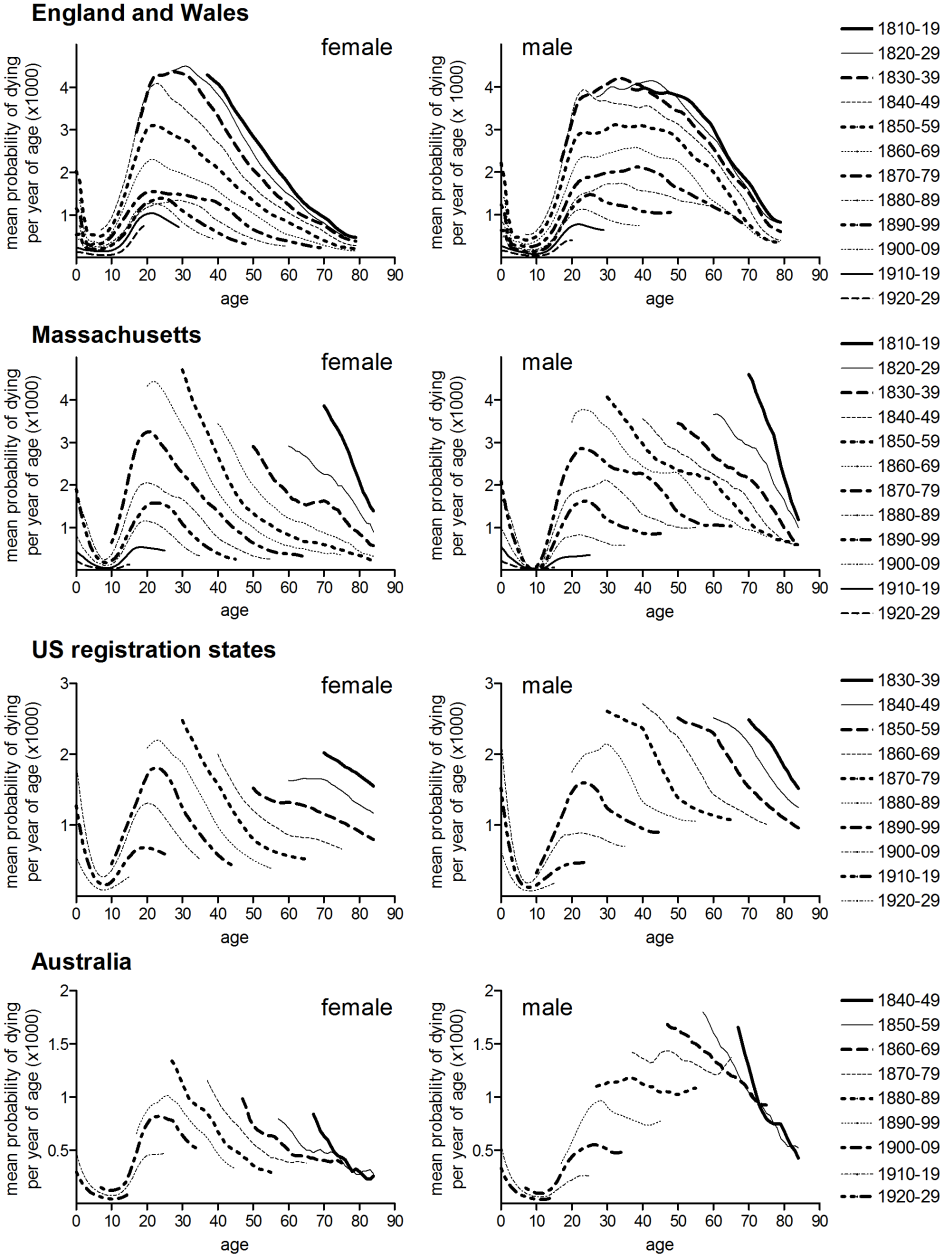
Age-specific tuberculosis mortality by birth cohort. Respiratory tuberculosis (England & Wales, and Massachusetts), all tuberculosis (US death registration states, Australia), plotted by ten year birth cohorts. Legend indicates cohort years of birth. Rates for years after 1945 were excluded.

**Figure 5:**
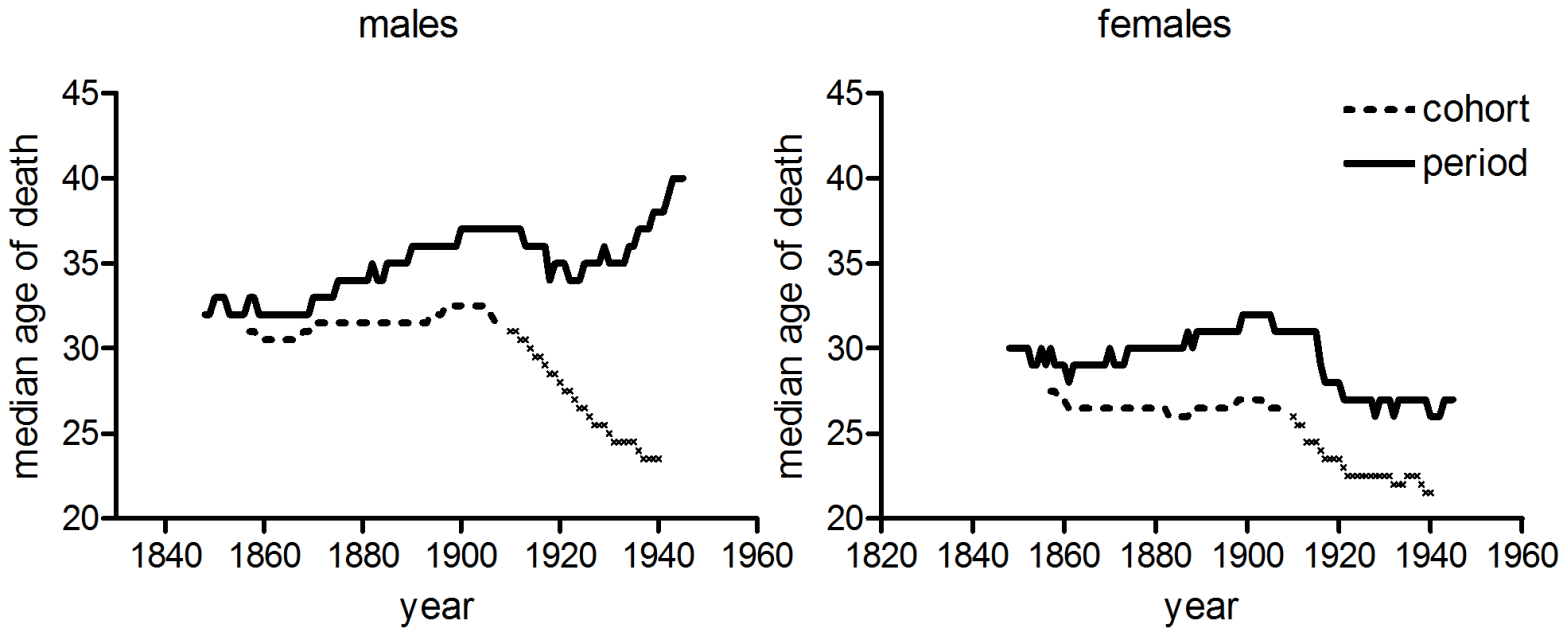
Median age at death from tuberculosis, by annual birth cohort, England and Wales. Median age was calculated by indirect standardisation of deaths at ages 10–79 years, and plotted at the year when the cohort was aged 20.

Figures 4–5 confirm the relative stability of age patterns by birth cohort compared with period measures. Figure 4 shows data for four populations where mortality rates could be constructed for well-defined consecutive birth cohorts. Compared with period rates (Figure 1), the cohort age distribution of mortality was more stable over time in both male and females, although the difference between period and cohort was slight for females and large for males. This is demonstrated for England and Wales in Figure 5, where median age at death is compared by cohort and period. Median age at death was almost invariant for birth cohorts born before 1890 (values for cohorts are plotted in Figure 5 according to the year when the cohort was aged 20, which provides a better comparison with period rates for the same years). As Spicer noted, cohort age patterns in England and Wales were relatively stable only in cohorts born before 1900 [49] (Figure 4). In the present case the progressive reduction in median age of death of cohorts born after 1890 evident in Figure 5 was a function of the dramatic declines in tuberculosis mortality after 1945, and therefore cannot be considered a ‘cohort effect’ with respect to patterns of tuberculosis mortality before antibiotic therapy. For this reason time trends in cohort data were not analysed statistically. By contrast with the stability of median age at death in older birth cohorts, period median age at death rose progressively from around 1870 in males, although this trend was temporarily reversed during WWI and in the aftermath of the influenza pandemic of 1918/19. In females median age at death was stable by both period and cohort before the second decade of the twentieth century. While subsequent falls in female median age were superficially similar by both period and cohort, falls in the cohort measure were driven by post-1945 falls in tuberculosis mortality, while the fall in period data may reflect the selective elimination of tuberculosis sufferers by pandemic influenza [50], [51].

The constancy of the age pattern of cohort rates compared with period rates constitutes the most compelling evidence for cohort effects in the literature, and has often been interpreted to mean that the age pattern of tuberculosis mortality was biologically determined [8], [20], [52], [53]. However while there was considerable uniformity in age patterns of mortality in female cohorts between populations (Figure 4), there was less consistency amongst male cohorts, suggesting that the factors determining age-specific rates were population-specific, at least for males [18], [22], [23].

### Timing of the Onset of Decline in Tuberculosis Mortality

**Figure 6:**
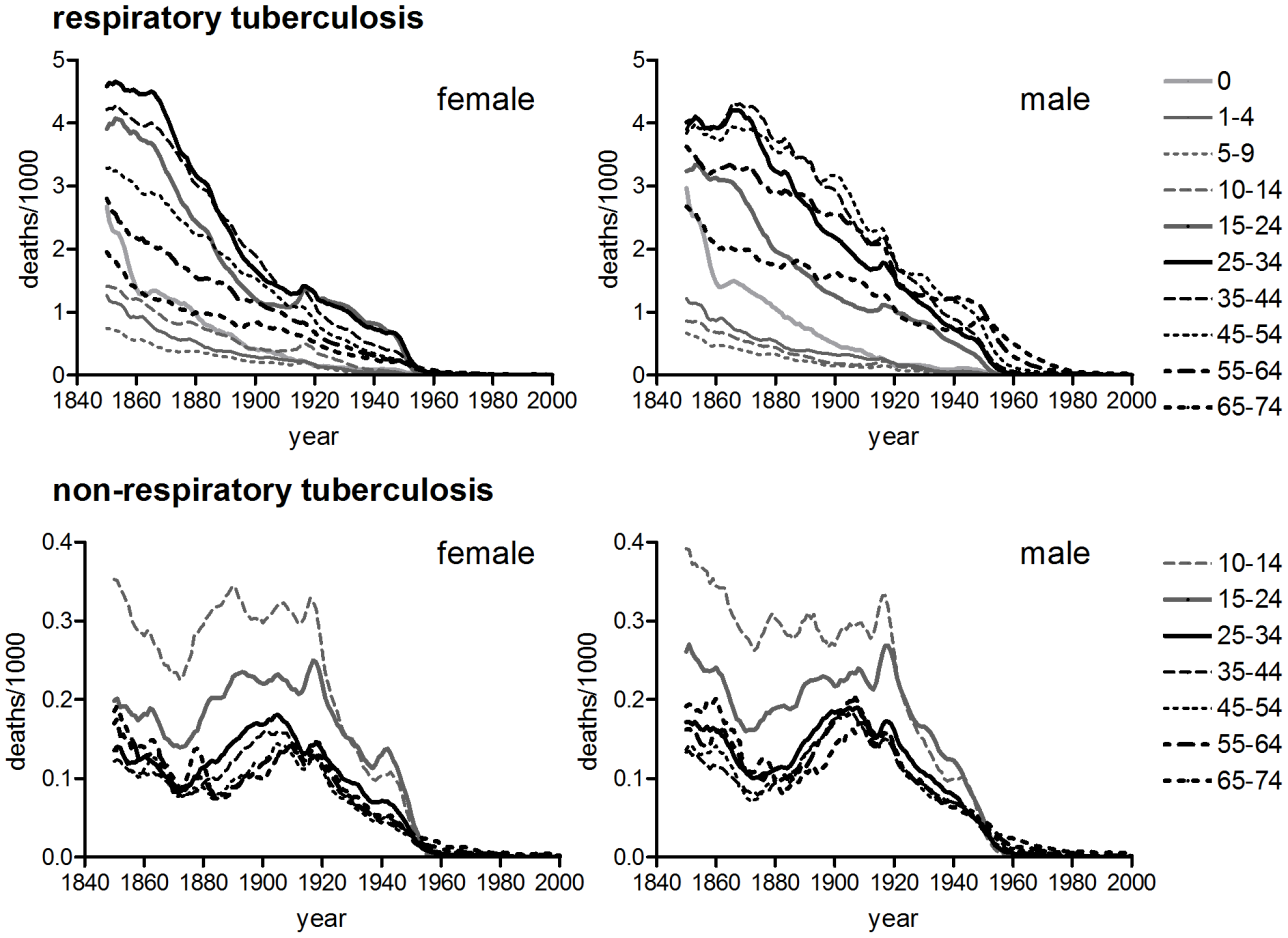
Age-specific respiratory and non-respiratory tuberculosis mortality in England and Wales, by period of death. Rates of non-respiratory tuberculosis at ages 0–9 were much higher than ages 10+, so were excluded to make the patterns in adulthood clearer.

**Figure 7:**
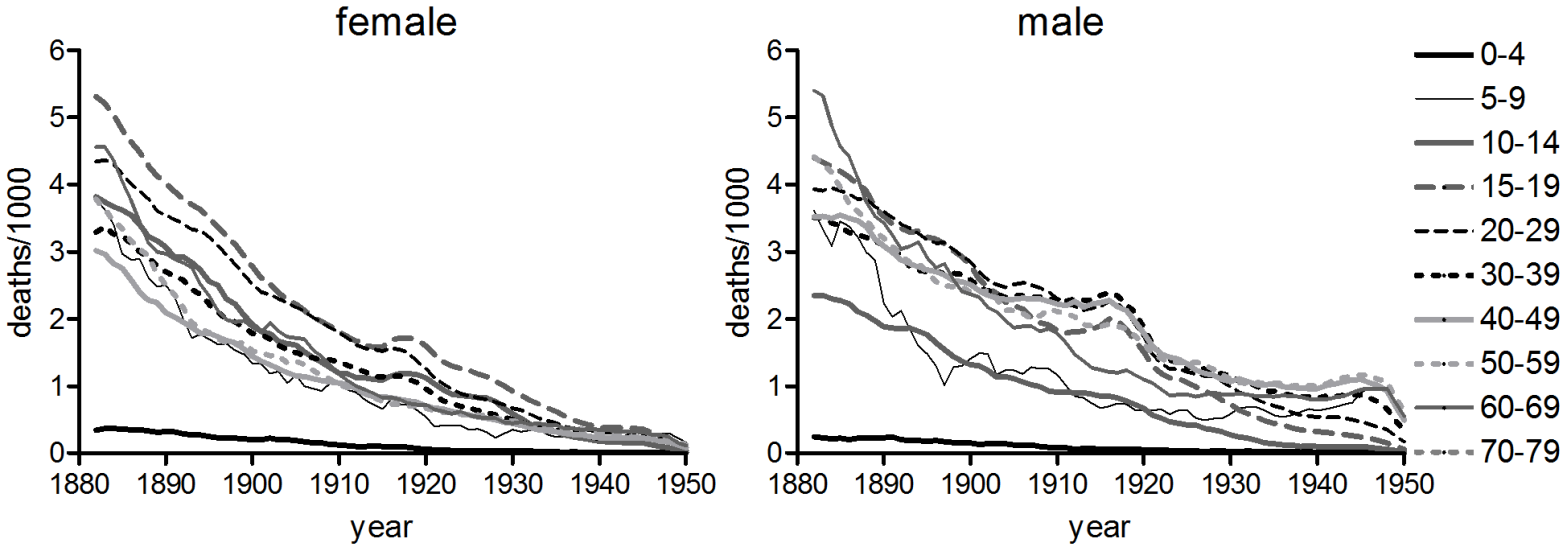
Age-specific tuberculosis mortality in Massachusetts, by period of death. Rates are for respiratory tuberculosis ages 0–14, and for all tuberculosis (predominantly respiratory) at ages 15+.

**Figure 8:**
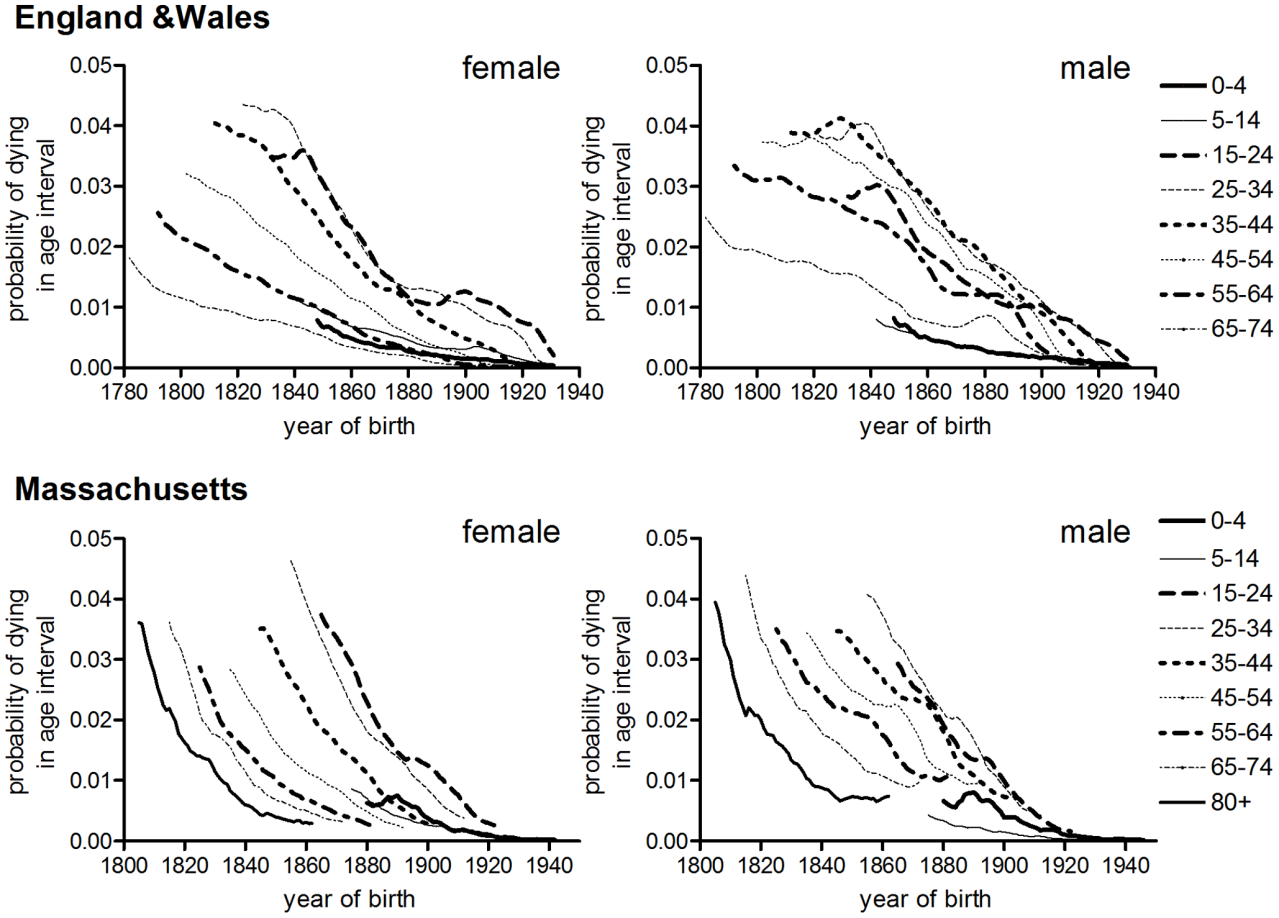
Age-specific respiratory tuberculosis mortality by birth cohort. Rates for years after 1945 were excluded.

The argument that susceptibility to tuberculosis was a property of birth cohorts and acquired early in life requires that decline should have occurred first in the youngest age groups, and then proceeded with progressive lags at successively older ages, as cohorts with lower risk progressed through life. Table 1 lists the populations that have been analysed for the presence of cohort effects in tuberculosis mortality. In most cases mortality decline was underway in multiple age groups from the beginning of the cause of death series, and so the hypothesis of age-dependent lags in mortality decline could not be tested. Even in the longest series available, for England and Wales, respiratory tuberculosis mortality appears to have been in decline in most age groups (with the notable exceptions of young adults of both sexes, and males aged 25–54) from the mid-nineteenth century (Figure 6). When mortality decline accelerated (in the 1870s in England and Wales, and 1880s in Massachusetts), it did so in multiple age groups simultaneously (Figures 6 and 7). Interestingly, in England and Wales the acceleration in decline of respiratory tuberculosis was paralleled by a rise in non-respiratory tuberculosis mortality, that was closely synchronised at all adult age groups (Figure 6). When age-specific respiratory tuberculosis rates were plotted by birth cohort for England and Wales, inflections in rates were not centred on any particular birth cohort, but were staggered by age in a manner typical of period effects (Figure 8). In Massachusetts, decline appeared to be underway in all birth cohorts born since the beginning of the nineteenth century (Figure 8).

**Figure 9:**
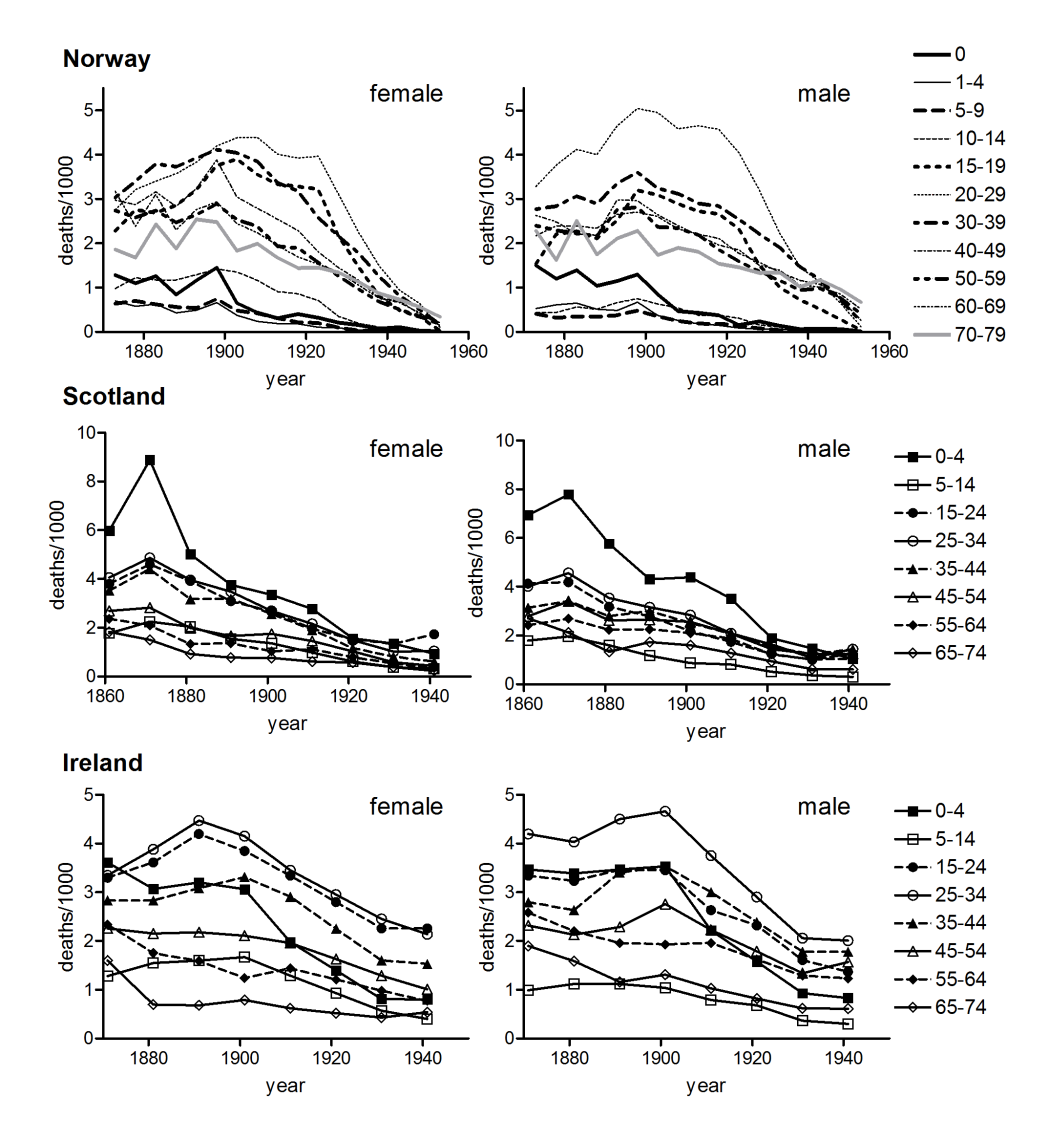
Age-specific tuberculosis mortality by period of death in populations with late declines. All tuberculosis (Ireland and Scotland), respiratory tuberculosis ages 0–14, all tuberculosis ages 15+ (Norway).

In three populations the onset of tuberculosis mortality decline occurred within observation (Figure 9). In each case the onset of mortality decline appeared to be simultaneous in multiple age groups (from c. 1900 in Norway and Ireland, and after 1870 in Scotland) indicating that the early phase of mortality decline was not confined to particular birth cohorts or age groups. Several definitions of onset of decline were used to test the simultaneity of decline (year on year decline for 8/10 years, decade in which 10% reduction first occurred), but both indicated that the onset of decline was coordinated in a broad range of age groups (Table 3). Similarly, analysis of percentage improvement in age-specific tuberculosis mortality rates using contour mapping [45] provided little evidence of cohort patterns in any of the populations with data amenable to this type of analysis (Figures S1–S5). There was some indication that improvement was most rapid in cohorts born after 1900 (indicated by the diagonal ‘staircase’ in the bottom right-hand corner of some plots). However most accelerations and decelerations in the rate of mortality decline tended to occur simultaneously across broad age ranges (indicated by the predominance of vertical patterns), even when the impact of the First World War and the 1918–19 influenza pandemic are discounted [50], [51].

**Table 3:** Dates of onset of decline of tuberculosis mortality in Norway, Scotland and Ireland.

	Norway^1^			
	male		female	
Age	8/10^2^	decade^3^	8/10^2^	decade^3^
0	1901–05	1901–05	1901–05	1901–05
1–4	1886–90	1886–90	1901–05	1901–05
5–9	1901–05	1901–05	1881–85	1881–85
10–14	1901–05	1901–05	1901–05	1901–05
15–19	1901–05	1901–05	1906–10	1906–10
20–29	1901–05	1901–05	1911–15	1911–15
30–39	1901–05	1901–05	1901–05	1906–10
40–49	1901–05	1901–05	1901–05	1901–05
50–59	1901–05	1901–05	1901–05	1901–05
60–69	1901–05	1876–80	1901–05	1901–05
70–79	1911–15	1911–15	1911–15	1896–1900
80+	1931–35	1901–05	1936–40	1876–80
	**Scotland** 4		**Ireland** 5	
	**male**	**female**	**male**	**female**
**age**	**decade** 3	**decade**	**decade**	**decade**
0–4	1872–81	1872–81	1902–11	1872–81
5–14	1872–81	1882–91	1902–11	1902–11
15–24	1872–81	1872–81	1902–11	1902–11
25–34	1872–81	1872–81	1902–11	1902–11
35–44	1872–81	1872–81	1902–11	1902–11
45–54	1872–81	1872–81	1902–11	1912–21
55–64	1872–81	1862–71	1872–81	1872–81
65–74	1862–71	1862–71	1872–81	1872–81
75+	1872–81	1872–81	1872–81	1872–81

–14, all tuberculosis ages 15+, measured over five year periods 1871–1955.^1^ Data refer to respiratory tuberculosis ages 0

‘8/10’ refers to the first period of a sequence of ten five year periods in which there was a decline recorded in eight or more periods.^2^

3 Decade in which mortality first fell by 10% or more (either the first five year period of the decade (for Norway), or the ten year period between the years of recorded mortality (for Scotland and Ireland)).

4 Data refer to all tuberculosis, reported for single years at decadal intervals 1861–1941.

5 Data refer to all tuberculosis, reported for single years at decadal intervals 1871–1941.

### Sex Differences in Age-specific Tuberculosis and Lung Cancer Mortality

**Figure 10:**
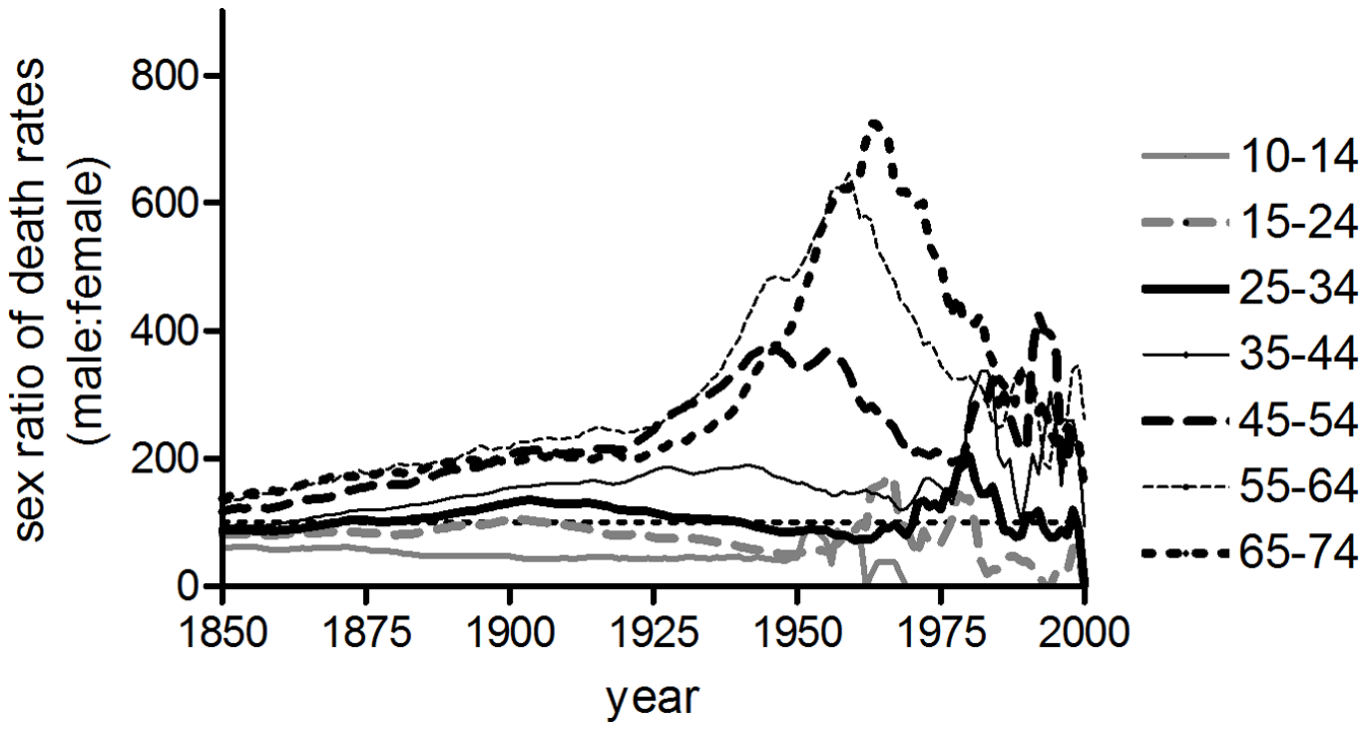
Age-specific sex ratios of respiratory tuberculosis mortality by period of death, England and Wales.

**Figure 11:**
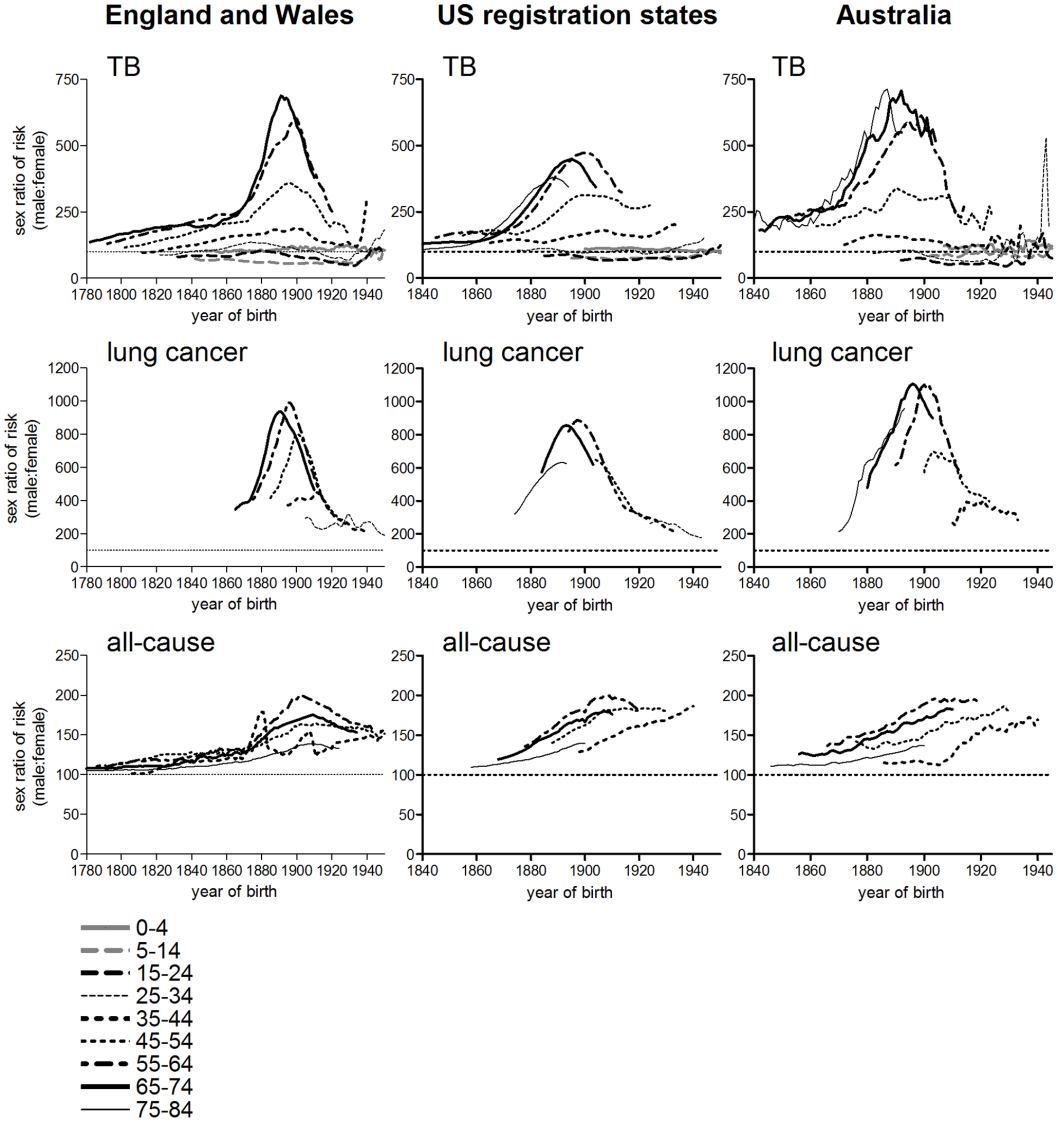
Age-specific sex ratios of tuberculosis, lung cancer and all-cause mortality, by birth cohort: England and Wales, USA and Australia. Plots represent all tuberculosis mortality (US, Australia) or respiratory tuberculosis (England & Wales). Data cover the periods described in Table 4.

**Figure 12:**
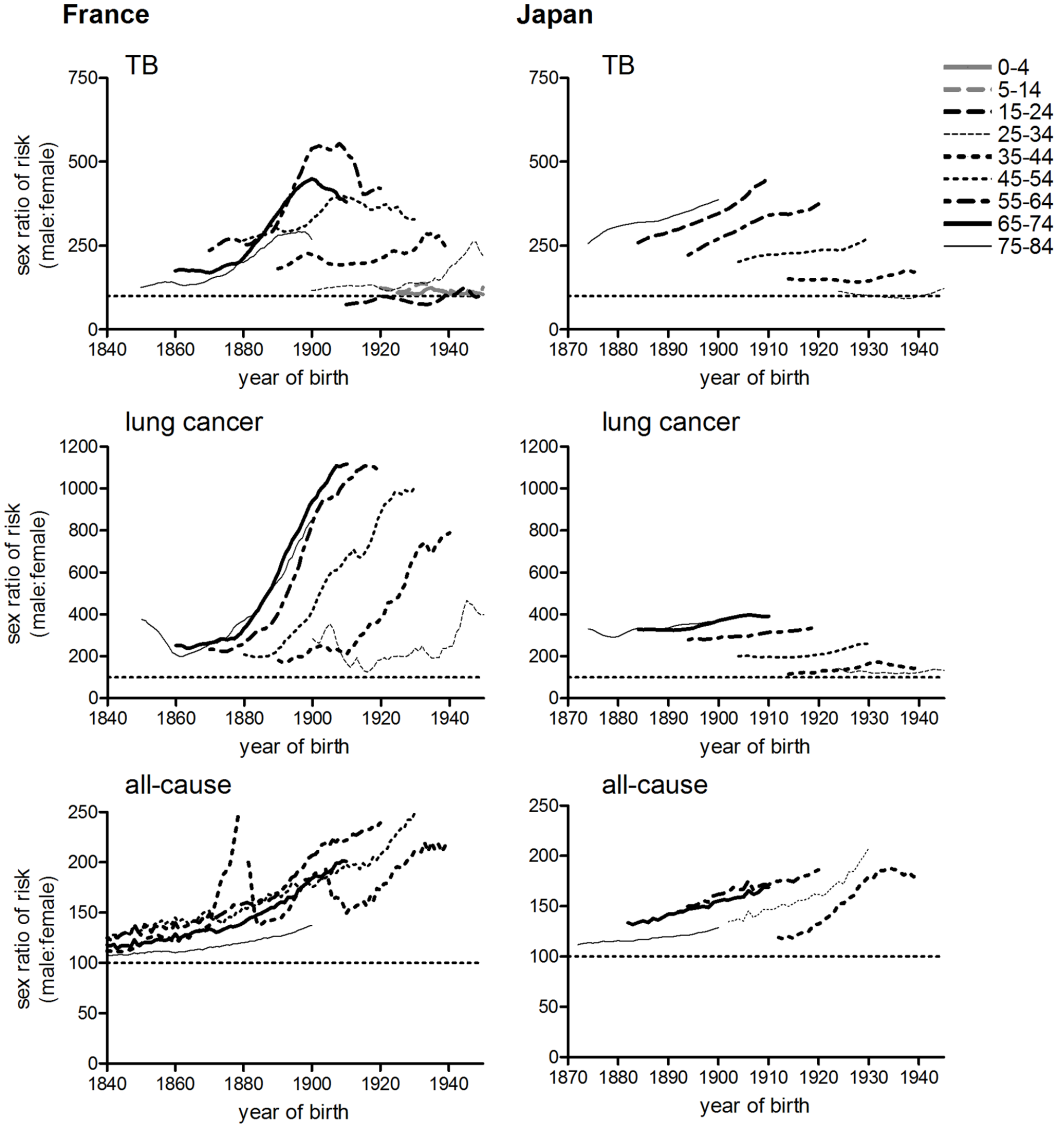
Age-specific sex ratios of tuberculosis, lung cancer and all-cause mortality, by birth cohort: France and Japan. Plots represent all tuberculosis mortality (France) or respiratory tuberculosis (Japan). Data cover the periods described in Table 4.

In most populations studied the age pattern of period female tuberculosis mortality remained relatively stable until the 1940s, whereas the age distributions for males shifted progressively to the right (Figures 1, 2 and 3). This produced an increasing disparity between the sexes in mortality rates in late adulthood. Mortality fell at roughly similar rates by sex at ages 0–24, and females remained at a disadvantage at ages 10–24 throughout the period up to 1980 (the sex ratio series shown in Figures 10, 11 and 12 include the period after the introduction of antibiotics, since large sex differences persisted despite dramatic falls in mortality; series were terminated in 1980 as rates became too low to measure the sex ratio reliably). However male disadvantage at ages 25 and older rose with age and over time, before falling again in a wave-like pattern (shown for England and Wales in Figure 10). When the sex ratio of tuberculosis mortality was plotted on a cohort basis, then excess male risk at older adult ages reached a peak in cohorts born around 1900 in England and Wales, in a manner highly reminiscent of cohort patterns associated with lung cancer, and with lifetime smoking habits (Figure 11, [54]). Sex ratios of tuberculosis and lung cancer mortality also followed very similar patterns in US registration states and Australia (although cohort patterns were less evident in these cases, for both causes of death) (Figure 11). Correlations between sex ratios of tuberculosis and lung cancer mortality were high for these three countries, and higher than correlations between tuberculosis and all-cause mortality (Table 4). Smoking patterns are a major cause of sex differences in all-cause mortality in adults [55]–[56], but are the dominant cause of sex differences in lung cancer rates in these countries [55], [57], [58]. Data for France (1925–1980) and Japan (1950–1980) were also included (Figure 12) because these populations differed in their smoking patterns from the anglophone populations studied here [59], [60]. In these populations sex ratios of tuberculosis mortality were more closely correlated with sex ratios of all-cause rather than lung cancer mortality (Table 4). These differences in sex ratio patterns between Japan and France on the one hand and the anglophone populations on the other may reflect differences in the trajectories of tuberculosis before the late 1940s (and the anomalous relationship between smoking and lung cancer in Japan [58], [61]), but do indicate that factors in addition to smoking must be considered in any explanation of the relative slowness of the historical decline in male tuberculosis rates.

**Table 4:** Pearson co-efficients (r) of correlation between sex ratios of tuberculosis, lung cancer, and all-cause mortality.

age groups	tuberculosis vs. lung cancer	tuberculosis vs. all-cause	lung cancer vs. all-cause	N^1^
**England and Wales, 1933–80**				
30–44	0.29***(143)^2,3^	0.15 (143)	−0.10 (144)	
45–64	0.81***	0.58***	0.51***	192
65+	0.91***	0.72***	0.70***	144
**Australia, 1945–80**				
30–44	0.11 (77)	0.18 (81)	0.00 (103)	
45–64	0.44*** (140)	0.17* (140)	0.28*** (144)	
65+	0.34*** (143)	0.10 (143)	0.55*** (144)	
**USA, 1950–78**				
30–44	−0.02	0.36***	−.60***	406
45–64	0.87***	0.32***	0.08*	580
65+	091***	0.51***	0.65***	580
**France, 1925–80**				
30–44	0.47***	0.34***	0.33***	168
45–64	0.69***	0.69***	0.94***	224
65+	0.86***	0.93***	0.89***	224
**Japan, 1950–80**				
30–44	0.30**	0.32**	0.35***	93
45–64	0.71***	0.76***	0.51***	124
65+	0.41***	0.58***	0.40***	124

1 Where N differed by cause (because of zero mortality rates), N is given in brackets with coefficient.

2 Asterixes indicate P<0.001 (***), P<0.01 (**), P<0.05 (*). The highest correlation between tuberculosis and lung or all-cause mortality is indicated in italics for each age group.

3 Non-parametric analysis using Spearman correlations produced very similar r (Rho) and P values in all cases.

## Discussion

Tuberculosis was the ‘white plague’ of the nineteenth century but declined dramatically in the decades before 1946, before the intervention of effective drug therapy or vaccination. This decline remains poorly understood, and has been variously attributed to increased human resistance due to genetic adaptation (see [62] for references); increased resistance arising from improvements in nutrition and poverty reduction (e.g. [63], [64]), reduced transmission, arising from confinement of cases or reductions in over-crowding (e.g. [24], [65]–[67]); and reduced virulence of the pathogen (e.g. [68]). Evidence of cohort patterns of mortality decline would constrain the range of possible causes of the decline, and could suggest a dominant role for re-activation in adult disease even in high prevalence settings. However re-examination of data previously presented in support of cohort patterns in tuberculosis mortality does not provide convincing evidence that tuberculosis mortality declined in a cohort-specific manner.

Tuberculosis decline began in most of the populations studied before the development of vital registration systems, and this makes it difficult to establish the existence or otherwise of the kind of lagged age-dependent initiation of mortality decline that Andvord considered diagnostic of cohort patterns. However in three cases where the initial stages of tuberculosis mortality decline occurred within observation the decline appeared to be initiated simultaneously in multiple, if not all, age groups (Figure 9, Table 3). Where tuberculosis was already in decline, then points of inflection in rates appeared to be co-ordinated in a wide range of ages (Figures 6, 7 and 8). Such simultaneity of changes across age groups is strongly suggestive of influences specific to the period, and independent of birth cohort.

The intuitively most compelling aspect of the cohort decline of tuberculosis hypothesis is the observed regularity of cohort age patterns of mortality (Figure 4), which suggests some underlying biologically determined age pattern of susceptibility. However although cohort age patterns were fairly similar amongst different female populations, cohort age patterns differed markedly between male populations, suggesting that the factors influencing age patterns were complex and perhaps sex-specific. Importantly, conservation of cohort age patterns could occur under conditions of either period- or cohort-dependent mortality decline. As long as decline was occurring at all ages, then the relatively high rates of mortality early in life would tend to maintain a youthful mortality peak in cohort rates, because as cohorts aged the survivors would live through progressively more benign mortality conditions. Thus Vynnecky and Fine were able to simulate cohort age patterns of tuberculosis mortality in England and Wales using very simple assumptions of age-independent decline and age-dependent susceptibility [9].

The second line of evidence for cohort effects, the progressive shift to the right of the male age distribution of tuberculosis mortality observed in some populations, did not constitute strong evidence of a cohort effect in the absence of evidence for a late onset of decline in these age groups. The absence of this shift in females, despite similar childhood mortality rates, makes it very unlikely that this pattern reflects effects of early life conditions on tuberculosis susceptibility. Rather these patterns arose from the relative slowness, but not lateness, of mortality decline at older ages in males of some populations. The widening of sex differences in tuberculosis risk over the first half of the twentieth century resembles closely the rise in excess male risk in mortality more generally in this period, and in particular the excess risk associated with smoking. Smoking grew rapidly in popularity in the late nineteenth century and in populations such as England and Wales, Australia and the USA its health impact was greatest in male cohorts born in the early twentieth century. Smoking is a risk factor for tuberculosis [69], [70], and smoking behaviours may be the primary cause of contemporary sex differences in tuberculosis notification rates [71]. Tuberculosis mortality became highly concentrated in older adults after the advent of effective chemotherapy, and this may reflect a rise in the relative importance of smoking as a risk factor, either for reactivation or disease progression after infection.

Previous claims regarding cohort patterns in tuberculosis mortality decline suggested a key role for infection in early childhood in determining lifelong risk, through primary disease in childhood and reactivation in adulthood. In contrast, the evidence presented here has no direct implications for debates regarding the importance of re-infection versus reactivation, or changes in resistance versus exposure, because the patterns reported (simultaneous declines in tuberculosis mortality at multiple ages) could be produced by a wide variety of factors, that could have affected re-infection and reactivation simultaneously, and could have improved resistance and/or exposure at most ages. Nevertheless the finding that tuberculosis mortality decline was not cohort-dependent has important implications for research into the distal causes of tuberculosis mortality decline. In this respect the most important function of this paper is to describe more clearly which patterns need to be explained. In particular any mechanisms proposed to account for declines in tuberculosis mortality should account for the simultaneity of changes in age groups predicted to differ in their history of exposure and immune status.

One of the most striking results of the present analysis is the diversity of patterns of historical tuberculosis decline. Tuberculosis mortality fell in most now-developed countries for decades before effective chemotherapy, but the timing of onset, and patterns of decline by age and sex, show marked differences between populations. This diversity presents a rich opportunity to test theories regarding the causes of tuberculosis mortality decline. Comparative modelling exercises should consider age-specific interactions that allow potential influences such as cohort-specific adult smoking habits and historical migration rates to be taken into account. Mason and Smith identified immigration as an important factor in the cohort patterns they discerned in US data, and speculated that mortality decline may have depended in part on a reduction in the proportion of foreign-born in the US population [25]. While Mason & Smith proposed that immigrants were less healthy than the locally-born, immigrants may also have been healthier on average than those remaining in the countries of origin. The exceptionally young age distribution of tuberculosis mortality in Norwegian males (Figure 2) was mirrored in the highly unusual age pattern of male all-cause mortality in the period 1856–1930, when mortality at ages 20–29 exceeded that at ages 30–39 [72]. These phenomena may reflect a negative selection effect of the very high emigration rates of young adult males in this period, which could have left the less robust to constitute a higher proportion of the population in the age group 20–29 than at other ages or amongst females. The impact of emigration may explain the late decline of tuberculosis in young adults in Norway (Figure 9) that seems to have formed the basis for Andvord’s perception of a cohort pattern in the Norwegian data. Age- and sex-specific migration patterns and associated ‘selection effects’ such as emigration of fitter individuals may explain some of the differences in age patterns between populations observed in Figure 1, and between historical urban and rural populations reported previously [22], [73]–[75].

## Conclusions

The patterns of decline of recorded tuberculosis mortality before the mid-twentieth century lend support mainly to theories of improvements that were relatively independent of age or prior exposure. The historical decline of tuberculosis therefore offers little evidence of benefits accruing to particular birth cohorts as a consequence of improvements in living conditions in childhood. The evidence for proportional hazard-type cohort patterns in tuberculosis mortality decline rests largely on the visual appeal of graphs showing cohort data for isolated years in males of selected populations with distinctive patterns of mortality. When more complete datasets for a range of populations and both sexes are considered then the evidence for this type of cohort effect appears very weak. Tuberculosis mortality decline is a poor example of a ‘cohort effect’ and should be dropped from textbooks in favour of other examples of cohort patterns such as lung cancer, where there is both better evidence and a biologically plausible explanation for the pattern. However the pattern of tuberculosis mortality decline is clearly complex and may involve both factors acting with immediate effect and factors with a prolonged or delayed effect on exposed groups that give rise to cohort-type patterns. Older males benefited less than other groups from the general decline in tuberculosis risk, and this probably reflects the contribution of a very well-established cohort pattern, that of smoking history and associated lung damage. Together with patterns of HIV infection, smoking habits may be a major influence on contemporary age distributions of tuberculosis morbidity, that has substantially modified the pattern of excess risk in early adulthood that predominated before 1950. This historical pattern of young adult risk remains poorly understood, but may provide important insights into age-specific aspects of tuberculosis immunity in contemporary populations.

## Supporting Information

Figure S1 **Annual percentage improvement in age-specific respiratory tuberculosis mortality rates, England and Wales 1848–1980.** Colours represent the range in percentage annual change indicated by the scale. Diagonal lines represent birth cohorts. Vertical patterns indicate that rates of mortality improvement were similar across age groups in a given year; diagonal patterns indicate that rates of change were more consistent within birth cohorts than by period.
(TIF)

Figure S2 **Annual percentage improvement in age-specific respiratory tuberculosis mortality rates, Massachusetts 1880–1950.** Five year age groups were used for ages 0–9, creating a discontinuity with single year of age data at older ages. Colours represent the range in percentage annual change indicated by the scale. Diagonal lines represent birth cohorts. Vertical patterns indicate that rates of mortality improvement were similar across age groups in a given year; diagonal patterns indicate that rates of change were more consistent within birth cohorts than by period.
(TIF)

Figure S3 **Annual percentage improvement in age-specific respiratory tuberculosis mortality rates, US death registration states, 1900–1980 (includes all US states from 1933).** Four-year age groups were used for ages 1–5, creating a discontinuity with single year of age data at older ages. Colours represent the range in percentage annual change indicated by the scale. Diagonal lines represent birth cohorts. Vertical patterns indicate that rates of mortality improvement were similar across age groups in a given year; diagonal patterns indicate that rates of change were more consistent within birth cohorts than by period.
(TIF)

Figure S4 **Annual percentage improvement in age-specific respiratory tuberculosis mortality rates, Australia, 1907–1960 (rates were too low and variable after 1960 to calculate meaningful rates of change).** Five year age groups were used for ages 0–4, creating a discontinuity with single year of age data at older ages. Colours represent the range in percentage annual change indicated by the scale. Diagonal lines represent birth cohorts. Vertical patterns indicate that rates of mortality improvement were similar across age groups in a given year; diagonal patterns indicate that rates of change were more consistent within birth cohorts than by period.
(TIF)

Figure S5 **Quinquennial percentage improvement in age-specific respiratory tuberculosis mortality rates, Norway, 1871–1955.** Colours represent the range in percentage annual change indicated by the scale. Diagonal lines represent birth cohorts. Vertical patterns indicate that rates of mortality improvement were similar across age groups in a given year; diagonal patterns indicate that rates of change were more consistent within birth cohorts than by period.
(TIF)

Data S1 **Deaths registered from tuberculosis in Massachusetts 1880–1950, documentation file.**
(DOCX)

Data S2 **Deaths registered from tuberculosis in Massachusetts 1880–1950 by age group and sex.** Comma-delimited text file as described in Data S1.
(TXT)
