# Supplementary figures and images for: Year of Birth Effects in the Historical Decline of Tuberculosis Mortality: A Reconsideration
Source: PLoS One. 2013 Dec 11;8(12):e81797. doi: 10.1371/journal.pone.0081797 (PMC3859563; doi:10.1371/journal.pone.0081797)

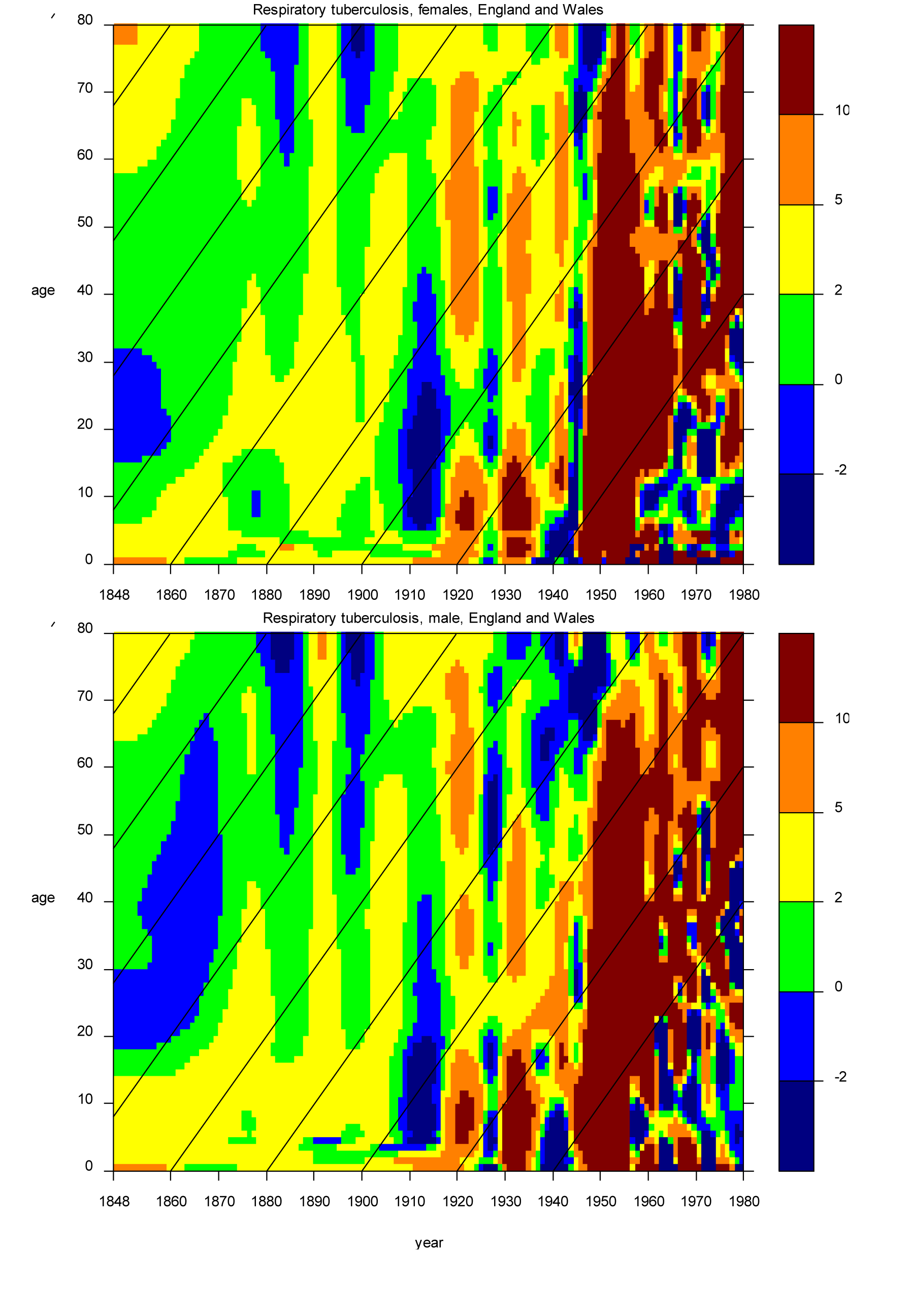

Supplement: Figure S1 — Annual percentage improvement in age-specific respiratory tuberculosis mortality rates, England and Wales 1848–1980. Colours represent the range in percentage annual change indicated by the scale. Diagonal lines represent birth cohorts. Vertical patterns indicate that rates of mortality improvement were similar across age groups in a given year; diagonal patterns indicate that rates of change were more consistent within birth cohorts than by period. (TIF) [file pone.0081797.s001.tif]

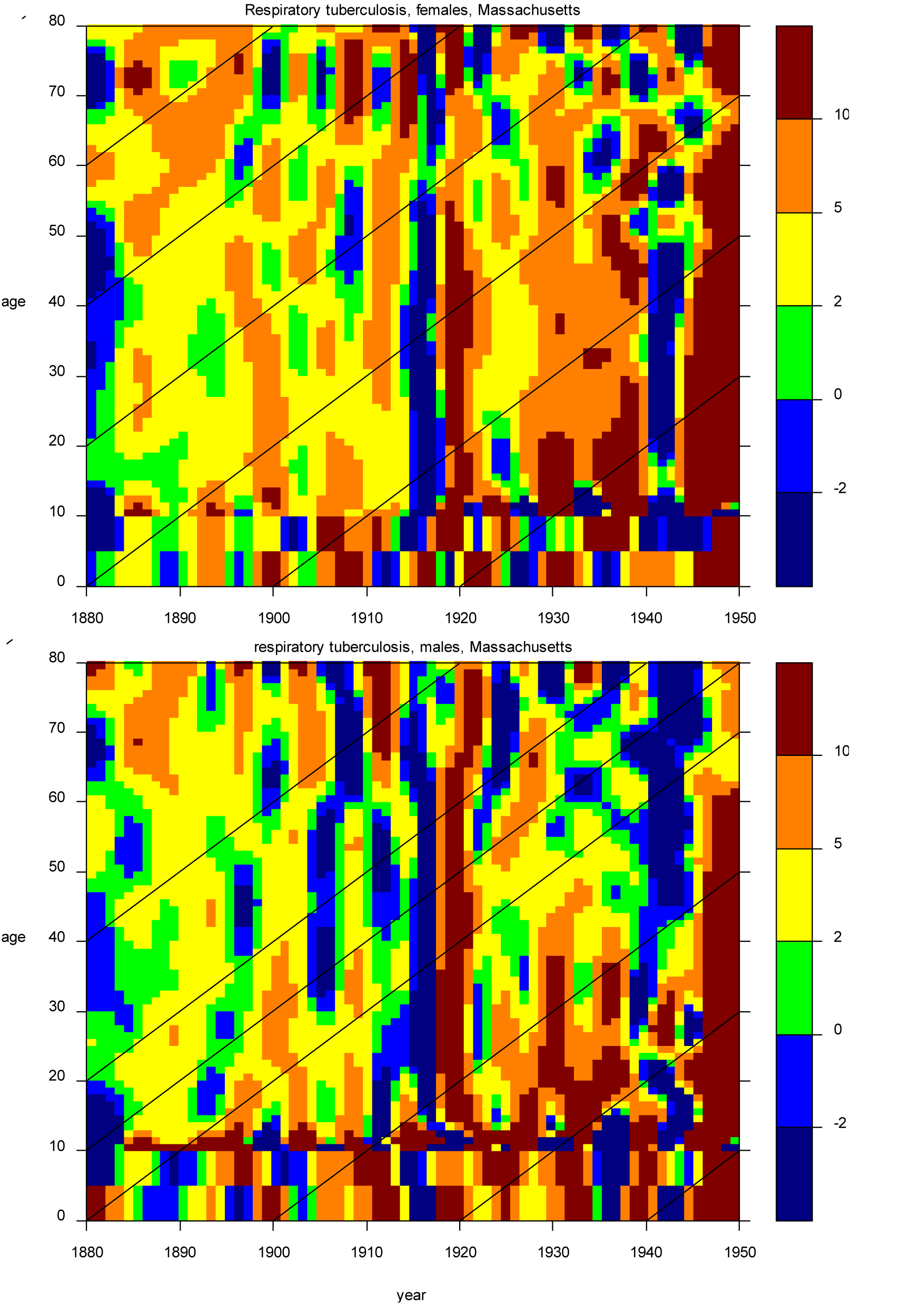

Supplement: Figure S2 — Annual percentage improvement in age-specific respiratory tuberculosis mortality rates, Massachusetts 1880–1950. Five year age groups were used for ages 0–9, creating a discontinuity with single year of age data at older ages. Colours represent the range in percentage annual change indicated by the scale. Diagonal lines represent birth cohorts. Vertical patterns indicate that rates of mortality improvement were similar across age groups in a given year; diagonal patterns indicate that rates of change were more consistent within birth cohorts than by period. (TIF) [file pone.0081797.s002.tif]

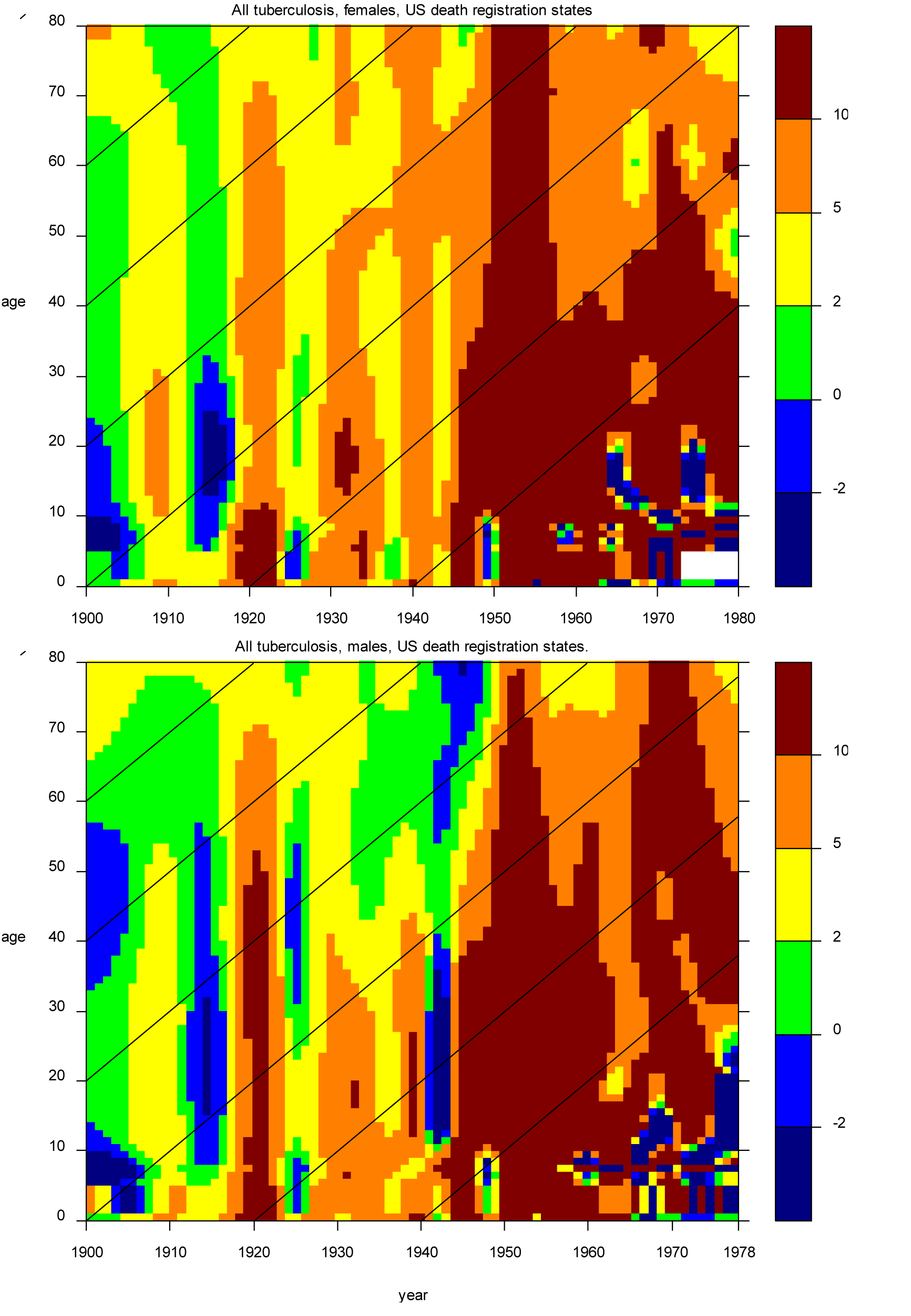

Supplement: Figure S3 — Annual percentage improvement in age-specific respiratory tuberculosis mortality rates, US death registration states, 1900–1980 (includes all US states from 1933). Four-year age groups were used for ages 1–5, creating a discontinuity with single year of age data at older ages. Colours represent the range in percentage annual change indicated by the scale. Diagonal lines represent birth cohorts. Vertical patterns indicate that rates of mortality improvement were similar across age groups in a given year; diagonal patterns indicate that rates of change were more consistent within birth cohorts than by period. (TIF) [file pone.0081797.s003.tif]

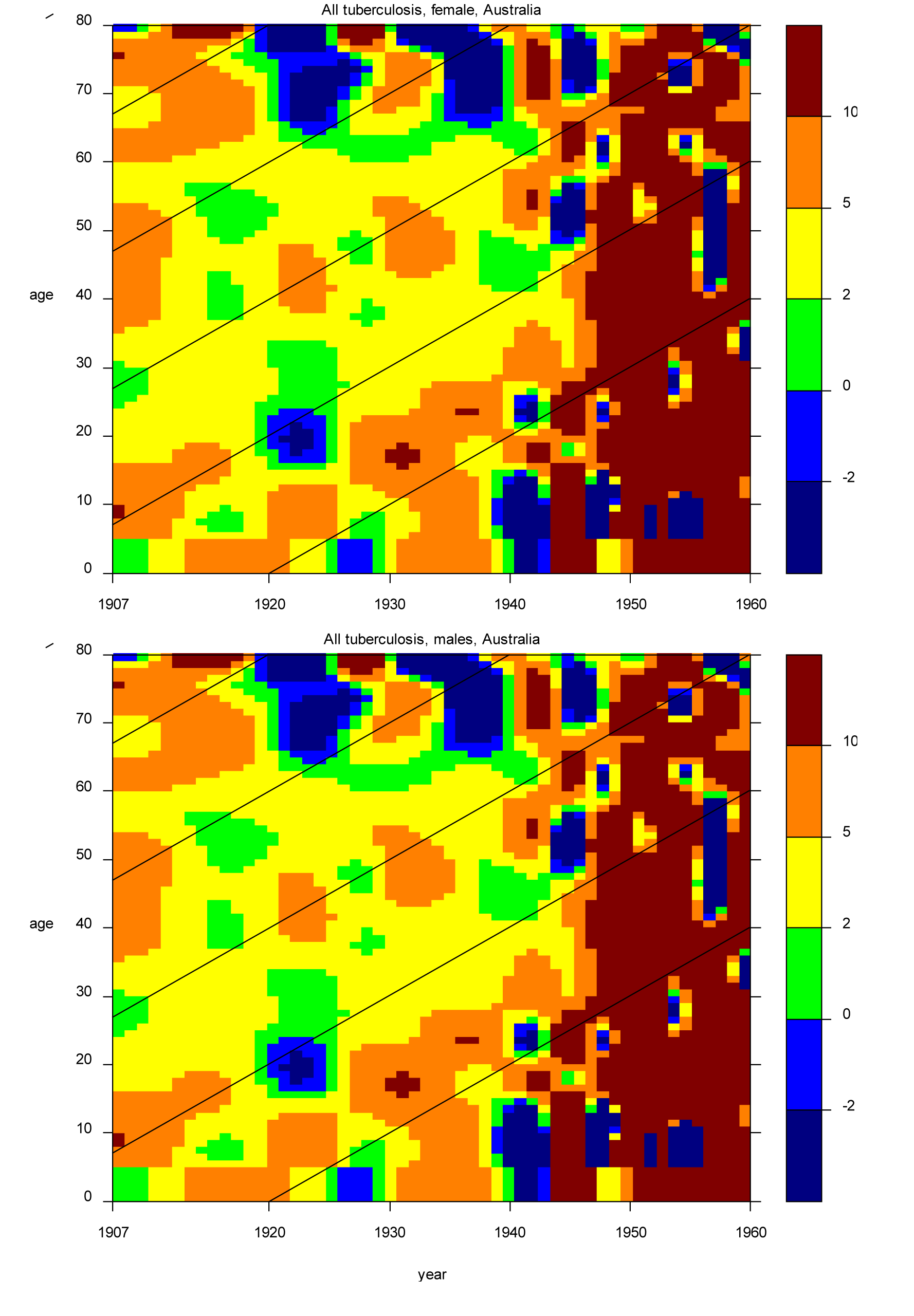

Supplement: Figure S4 — Annual percentage improvement in age-specific respiratory tuberculosis mortality rates, Australia, 1907–1960 (rates were too low and variable after 1960 to calculate meaningful rates of change). Five year age groups were used for ages 0–4, creating a discontinuity with single year of age data at older ages. Colours represent the range in percentage annual change indicated by the scale. Diagonal lines represent birth cohorts. Vertical patterns indicate that rates of mortality improvement were similar across age groups in a given year; diagonal patterns indicate that rates of change were more consistent within birth cohorts than by period. (TIF) [file pone.0081797.s004.tif]

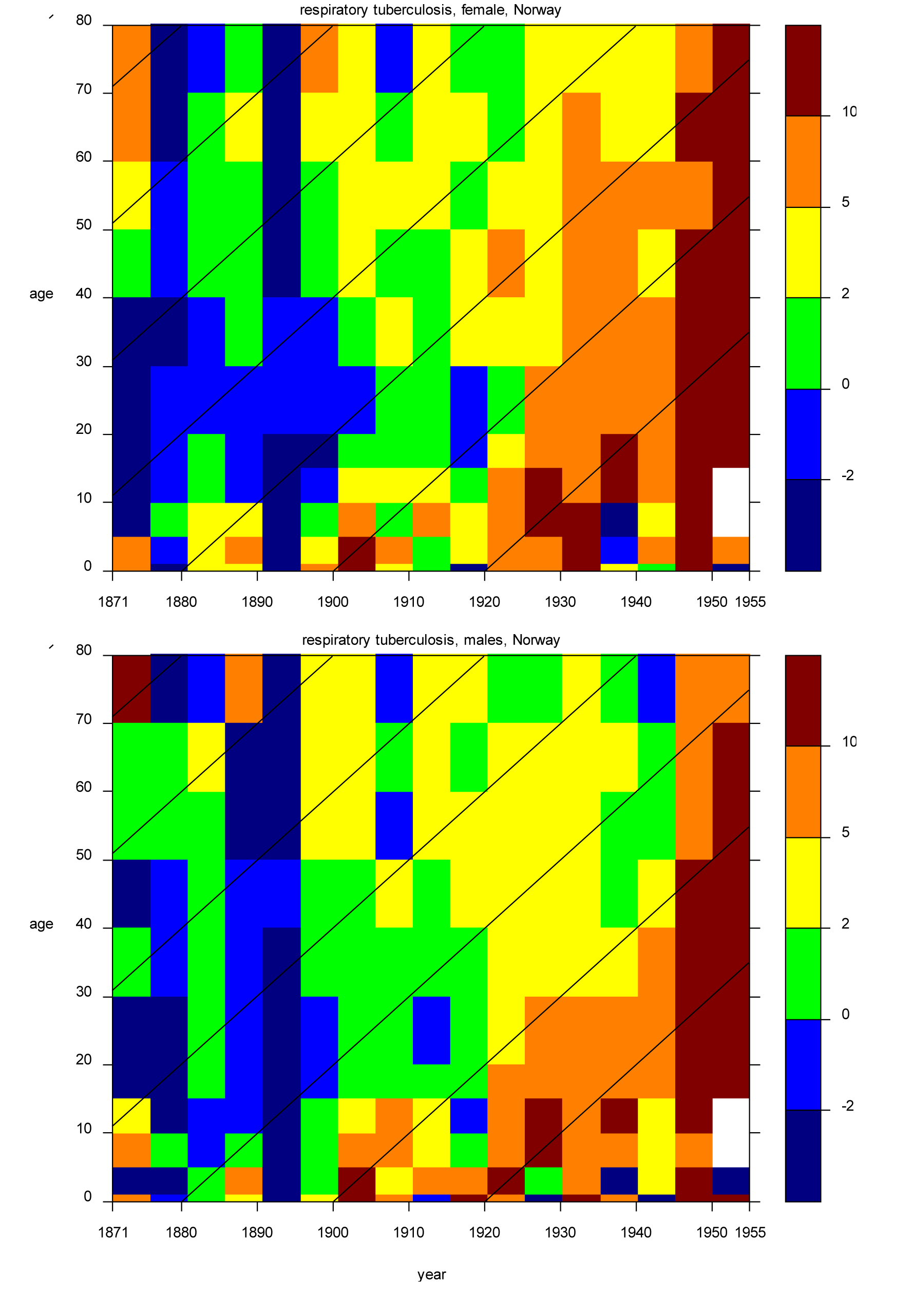

Supplement: Figure S5 — Quinquennial percentage improvement in age-specific respiratory tuberculosis mortality rates, Norway, 1871–1955. Colours represent the range in percentage annual change indicated by the scale. Diagonal lines represent birth cohorts. Vertical patterns indicate that rates of mortality improvement were similar across age groups in a given year; diagonal patterns indicate that rates of change were more consistent within birth cohorts than by period. (TIF) [file pone.0081797.s005.tif]
