# Supplementary material for: Year of Birth Effects in the Historical Decline of Tuberculosis Mortality: A Reconsideration
Source: PLoS One. 2013 Dec 11;8(12):e81797. doi: 10.1371/journal.pone.0081797 (PMC3859563; doi:10.1371/journal.pone.0081797)
Supplement: Data S1 — Deaths registered from tuberculosis in Massachusetts 1880–1950, documentation file. (DOCX) [file pone.0081797.s006.docx]

**Data S2. Deaths registered from tuberculosis in Massachusetts 1880-1950 documentation file**

Annual counts of deaths from tuberculosis by age-group and sex were transcribed from the *Annual reports of the vital statistics of Massachusetts* (title varies) for the years 1880-1950. The data are available as a comma-delimited text file (Data S2). Variables in the dataset are:

*year*: year of death as recorded in the *Annual Reports*

*code*: code assigned to cause of death in *Annual Reports* (varies by year)

*cause*: cause of death category in *Annual Reports* (varies by year)

*sex*: sex reported in *Annual Reports*

*total*: total deaths in cause of death category by sex

*a0*: deaths aged less than one year

*a1, a2, a3, a4*: deaths at age 1, 2, 3, 4

*a0-4*: deaths at ages 0-4 years, and similar for other age groups

*unknown*: deaths where age was not reported.

Note that the age groups used to report counts of deaths varied by period.
